# Effect of Texturing Environment on Wetting of Biomimetic Superhydrophobic Surfaces Designed by Femtosecond Laser Texturing

**DOI:** 10.3390/nano12183099

**Published:** 2022-09-07

**Authors:** Salomé Basset, Guillaume Heisbourg, Alina Pascale-Hamri, Stéphane Benayoun, Stéphane Valette

**Affiliations:** 1 Laboratory of Tribology and Systems Dynamics, Ecole Centrale de Lyon, 69130 Ecully, France; 2 EDF R&D—Lab Les Renardières, 77250 Ecuelles, France; 3 Manutech USD, 42000 Saint-Etienne, France

**Keywords:** biomimetics, wetting, superhydrophobicity, femtosecond laser, surface texturing

## Abstract

Inspired by *Euphorbia* leaves, micrometric pillars are designed on 316L stainless steel surfaces using a femtosecond laser to achieve superhydrophobicity. In this study, we focus on wetting behavior evolution as a function of time and chemical environment. Two types of texturing designs are performed: the laser texturing of micrometric square pillars, and the laser texturing of micrometric square pillars whose tops were irradiated using various fluences to obtain a different topography on the nanometric scale. Two laser texturing environments are considered in both cases: a CO_2_ flow and ambient air. The main result is that 250 days after laser texturing, steady-state contact angles (SSCA) were above 130° no matter what the environment was. We also study the effect of regular wetting over time. Comparing the results of surfaces for which wetting over time was conducted and that of the undisturbed surfaces for 250 days demonstrates that performing wetting measurements when the surface is not stable led to major changes in droplet behavior. Our surfaces have a unique wettability in which droplets are in an intermediate state. Finally, using a CO_2_ flow did not help reach higher SSCA, but it limited the effect of regular wetting measurements.

## 1. Introduction

A surface is superhydrophobic if it exhibits steady-state contact angles above 150° and very low hysteresis values [1] (below 20° and ideally below 10°) to ensure water-repellent properties. Two classical models can be distinguished to explain the wetting behavior of a superhydrophobic surface. In the Wenzel model [2], the droplet fully wets the surface and its asperities, while in the Cassie–Baxter model [3,4], the droplet stays on top of the asperities, leaving an air layer within them. There are also mixed states, and the most well-known is the rose petal configuration [5], in which the droplet is in the Wenzel state on the micrometric scale but the Cassie–Baxter state on the nanometric scale. This specific configuration is characterized by the high adhesion of water to the surface, leading to hysteresis of 180° with no droplet deformation upon inclination, and contrasts with the Lotus effect where the droplet easily rolls off the surface [6].

These superhydrophobic surfaces are found extensively in nature and have attracted attention over the past decades due to their wide range of potential applications, including in the prevention of biofilm development [7,8], corrosion [9], and ice formation [10]. Traditionally, water repellency is achieved by covering the desired surface with hydrophobic coatings [11,12,13,14,15,16,17,18,19]. Despite their high performance, these coatings present some serious drawbacks, such as the use of controversial chemistry that tends to be avoided nowadays [20,21]. More recently, nano-, pico-, or femtosecond laser texturing became a new pathway leading to the superhydrophobicity of surfaces with no impact on nature or humans [22,23,24,25]. Femtosecond laser texturing has the advantage of having greatly reduced heat-affected zones, no recast layer, nor micro-cracks or surface debris [26,27]. It is possible to proceed to the ablation of any kind of material such as metals [28,29], polymers [30,31], and even biological tissues [32,33], and generate micro- and nanostructures with high precision [34,35]. Moreover, the emergence of biomimicry helped to solve many complex problems, like the development of structures and materials for energy absorption applications [36], surfaces with improved erosion resistance inspired by desert scorpions [37], and a bacteria-based self-healing concrete [38], to cite a few examples. Therefore, it is interesting to gather femtosecond laser texturing and biomimicry to find new nature-inspired designs that can be reproduced on different materials [39,40,41,42,43], unlike the non-hierarchical structures reported by most papers regarding superhydrophobic surfaces [44,45,46,47,48].

It is important to note that superhydrophobicity in nature is not only reached by a specific multiscale topography but also by surface chemistry allowing a robust water repellency [49,50]. Laser texturing simultaneously changes the topography and the surface chemistry of the textured material. Consequently, it brings new challenges to overcome, such as the reactivity of the newly textured surface, as it is difficult to separate both contributions. When a sample is textured using a laser, its surface is hydrophilic or even superhydrophilic right after the process and gradually evolves to a hydrophobic regime over time [25,51,52]. This evolution might be due to the adsorption of organic compounds to the surface [53] or the dissociative adsorption of carbon dioxide from ambient air [25,52]. To accelerate this evolution it is possible to perform specific heat treatments [54] or to store the samples in a carbon-rich environment [52]. Most papers also report a post-chemical treatment with the immersion of the sample in solutions containing a silane reagent with fluoride [45,55,56], known to have hydrophobic properties, combining a specific topography and surface chemistry favorable to lower surface tensions leading to a strong superhydrophobicity.

**Figure 1:**
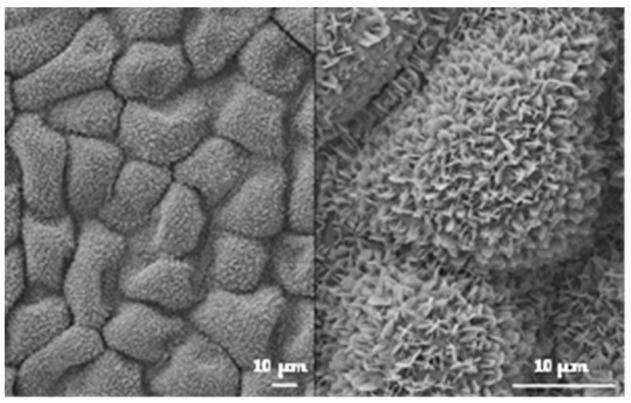
SEM images of a Euphorbia leaf. Credits: Maxime Bronchy—EDF R&D MMC 2017.

Kietzig et al. [52] found that storing newly femtosecond laser textured samples in a CO_2_-rich environment had a positive impact on the superhydrophobicity in terms of water contact angle and its corresponding time-evolution compared to the storage in ambient air or boiling water. The start of this study is the hypothesis that direct exposition to CO_2_ during the texturing process can improve the wetting properties of 316L and could lead to avoiding the use of any post-chemical treatment. In this work, we used femtosecond laser texturing to produce micrometric square pillars inspired by *Euphorbia* leaves (Figure 1) without post-treatment. For some samples, the top of the square pillars was irradiated using increasing fluences to obtain different topographies in the nanometric scale. Two texturing environments were considered: ambient air and a constant CO_2_ gas flow. The evolution of wetting over time was also carried out.

## 2. Materials and Methods

### 2.1. Femtosecond Laser Texturing Process

**Figure 2:**
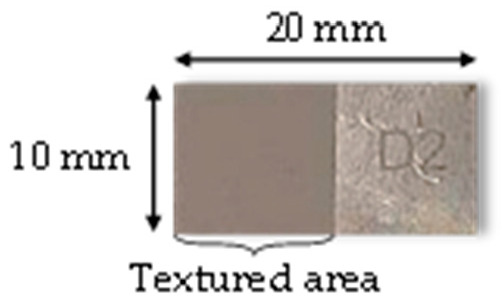
Dimensions of the 316L sample.

The materials considered are samples of pre-polished plates of 20 × 10 × 2 mm^3^ of 316L stainless steel (AMB, La Ferté Alais, France) with Ra = 0.4–0.8 µm. The samples were not polished further, and 1 cm^2^ was textured while the other cm^2^ was untreated and served as reference (Figure 2).

Before being textured, the surfaces were ultrasonically washed for 10 min in acetone to remove any organic compounds, followed by an ultrasonic bath of distilled water (10 min) and an ultrasonic bath of ethanol (10 min) to remove any residual water.

**Figure 3:**
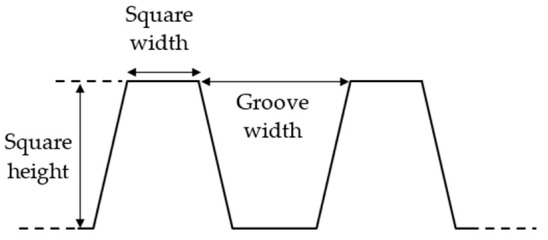
Dimensions of micrometric square pillars textured on the 316L sample.

**Figure 4:**
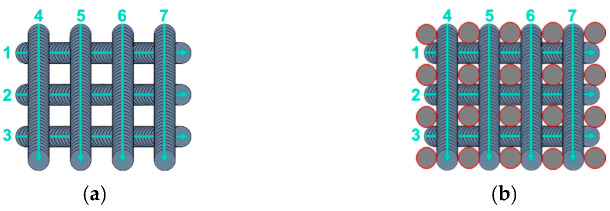
(**a**) Texturing process to generate micrometric square pillars; (**b**) texturing process to generate micrometric square pillars followed by irradiation of their tops.

To obtain micrometric square pillars having a height of 20 µm, a width of 10 µm, and a groove width of 20 µm (Figure 3), the samples were textured as described in Figure 4a using a 30 W FiberCryst femtosecond laser (Décines-Charpieu, France) with a central wavelength of 1030 nm, a pulse duration of 650 fs, a 325 kHz repetition rate, and a scan rate of 1.625 m/s. A lens with a focal length of 100 mm was used to focus the laser beam and get a beam diameter of 33 µm. The output power was decreased to 0.56 W to have a fluence of 0.20 J/cm^2^ and 150 overscans were necessary to achieve the desired square pillar dimensions.

For some samples, the top of the square pillars was irradiated using different fluences (0.5, 3.99, and 10.1 J/cm^2^), as demonstrated in Figure 4b. This irradiation was performed right after texturing the pillars to ensure optimum positioning of the laser beam on the top of the square pillars, thanks to the laser software. In this case, there was almost no laser beam coverage, and three pulses were performed on each top. These conditions were chosen to get an adequate range of fluences without damaging the micrometric scale of the textured pillars but still induce a local change in the nanometric scale. The samples for which the pillars’ top was irradiated are referenced according to the fluence used. The others are referenced as “no square pillars’ top irradiation”. Half of the samples were textured in ambient air, and the other half were textured under a constant CO_2_ flow (12 L/min) to study the effect of the environment on the laser process and wetting behavior. The CO_2_ flow was chosen as the dissociative adsorption of CO_2_ onto a newly textured surface is a possible explanation for its transition from hydrophilicity to hydrophobicity [52]. The high energy delivered by the femtosecond laser could accelerate this phenomenon. After the texturing process, the samples were stored in ambient air.

### 2.2. Sample Characterization

#### 2.2.1. Topography Analysis

The topography was qualitatively and quantitatively analyzed using a Scanning Electron Microscope (MIRA3 Tescan, Brno, Czechia) and a Bruker interferometer (Billerica, MA, USA), respectively. SEM analysis was performed using a secondary electron detector with an accelerating voltage of 10 kV and several optimal parameters to properly image the surfaces at different scales. This was useful to notice any visual difference between the samples textured in ambient air or under a CO_2_ flow. The SEM procedure was conducted once the surface chemistry was stable, not to perturb its chemical evolution [57].

Topography was then further studied quantitatively by optical interferometry in Vertical Scanning Interferometry (VSI) mode with a green light (wavelength of 515 nm), a Michelson objective (×5), and a ×2 lens. The patterns obtained were analyzed using the MountainsMap software developed by DigitalSurf (Besançon, France). The dimensions of the micrometric square pillars were verified, and arithmetic mean roughness (Ra) was calculated for the nanometric and micrometric scales considering ISO 4287 standards.

#### 2.2.2. Surface Chemistry Analysis by XPS

The surface chemistry of some samples was analyzed by X-ray photoelectron spectroscopy (XPS). The XPS used was an Ulvac-Phi Versaprobe II (Chigasaki, Japan) with a monochromatic Al Kα radiation (h*υ* = 1486.6 eV). Wide-scan and high-resolution spectra for C1s and O1s were obtained. The binding energy was calibrated with the C1s signal at 284.8 eV. The peaks were identified using the reference handbook of XPS [58].

#### 2.2.3. Contact Angle Measurements

**Figure 5:**
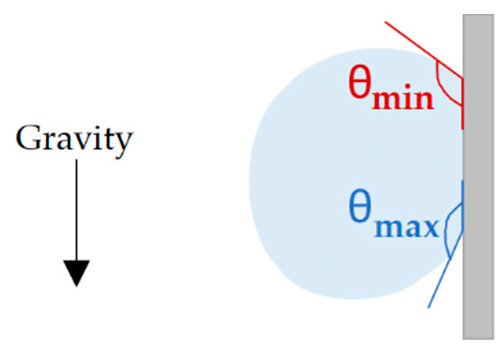
Schematic illustrating the measurement of θ_max_ − θ_min_ when the surface is tilted at 90° of inclination and the droplet is still anchored.

For each sample, steady-state contact angle measurements were carried out by depositing four 3 µL deionized water droplets on the stainless-steel surfaces using a DSA 30 Kruss goniometer. A video was recorded and θ_sessile_ was averaged after stabilization of the droplet on the surface. The surface was then tilted to 90° to measure θ_advancing_ and θ_receding_. These angles can define hysteresis if the droplet rolls off the surface. However, as we see in the Results and Discussion section, none of the droplets deposited on the textured samples rolled off the surface. They were still anchored to the surface at 90° of inclination. Therefore, we measured θ_max_ − θ_min_ at 90° of inclination. This value is a good indication of the droplet deformation upon inclination (Figure 5). Measurements were carried out at 0, 1, 2, 3, 17, 22, 28, 37, 43, 50, 77, 150, and 252 (or 254) days after being textured by the femtosecond laser. The first measurement was conduced a few hours after the laser texturing process. Before these measurements, the surfaces were blown using nitrogen gas to remove particles that could alter the droplet behavior.

## 3. Results and Discussion

### 3.1. Effect of Texturing Process Environment on Topography

**Figure 6:**
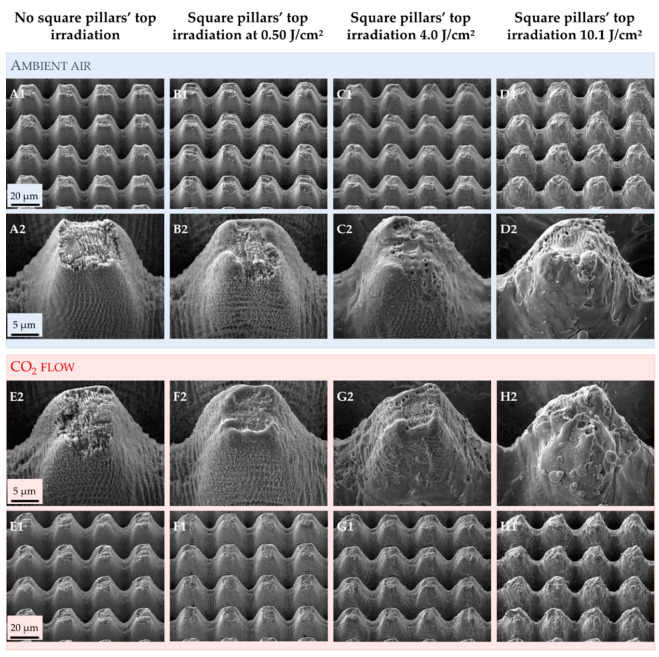
SEM images of the samples textured in ambient air or under a CO_2_ flow for which the top of the square pillars was irradiated using different fluences. Images from (**A**–**D**) are samples textured in ambient air. Images from (**E**–**H**) are samples textured under a CO_2_ flow. Images denoted with the number “1” are wide views of the textured area, while the number “2” is a focus on one square pillar from the wide view.

The micrometric square pillars were chosen as a simplified design matching the Euphorbia leaf morphology. The Euphorbia leaf, like many other hydrophobic plants, has a multi-scale topography, and reproducing the nanometric scale can be challenging. From the SEM images (Figure 6), we can see that we successfully generated a double-scale morphology on 316L similar to that of the Euphorbia leaf.

Several papers demonstrated that texturing in different environments could generate various structures [59,60]. The first step of this study was to ensure that the texturing atmosphere did not affect the topography, whether it be on the micrometric scale or the nanometric one. The SEM images (Figure 6) show that irradiating the top of the square pillars mainly affects the nanometric scale. When the top of the square pillars is not irradiated, the entire surface is covered by ripples (Figure 6A,E). When the top of the square pillars is irradiated at 0.50 J/cm^2^ (Figure 6B,F), smooth areas can be seen, and these smoother areas enlarge progressively when the fluence increases (Figure 6C,G) until almost no ripples can be spotted at 10.1 J/cm^2^ (Figure 6D,H). With increasing fluence, the deposition of molten matter appears more chaotic and shifts the nano-roughness of the ripples to a micro-roughness.

These smooth areas could be due to local surface fusion [61]. Even if femtosecond laser texturing is an almost athermal process, the laser beam diameter was wider than the square pillar width (33 µm vs. about 13 µm). Therefore, the grooves and slopes of the pillars were also irradiated. When the lowest fluence of 0.50 J/cm^2^ is irradiating the top of the square pillars, it only affects this location (Figure 6B,F). It is where the laser beam energy is the highest, and the residual energy of the Gaussian beam is too low to impact the roughness of the grooves and slopes. When higher fluences are used, the residual energy of the Gaussian beam is high enough to modify the roughness in the slopes and grooves.

Finally, the texturing environment does not seem to affect this evolution, as similar images are recorded for the samples textured in ambient air and under a CO_2_ flow. However, we can note that the square pillars seem to have more rounded tops when the surfaces are textured under a CO_2_ flow.

**Figure 7:**
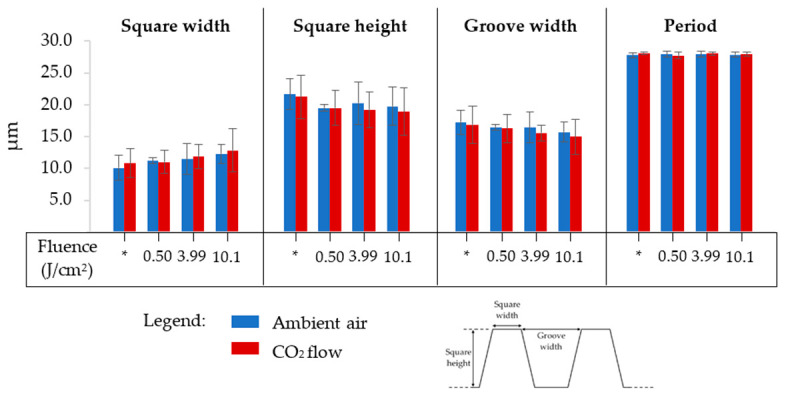
Dimensions of the micrometric square pillars designed on 316L SS for all the samples textured in ambient air or under a CO_2_ flow with irradiation of their tops at different fluences; * = samples for which the square pillars’ top was not irradiated.

Figure 7 summarizes the square pillars’ dimensions of all the samples considered in this study: square pillar width, square pillar height, groove width, and period (square pillar width + groove width). The texturing environment has no impact on these dimensions: the square pillars have a width and height of 13.0 ± 0.9 µm and 19.5 ± 1.3 µm, respectively, when textured in ambient air, and 12.9 ± 0.8 µm and 19.4 ± 1.2 µm, respectively, when textured under a CO_2_ flow. The grooves’ widths are equal to 15.5 ± 0.9 µm in ambient air and 15.2 ± 0.9 µm under a CO_2_ flow. Finally, the period is the same in both environments, ≈ 28.3 µm. We note a slight decrease in the square pillar height and an increase in square pillar width of about 2 µm in both cases upon rising the fluence. As can be seen in the SEM images in Figure 6, the slopes of the square pillars are not 90°. Therefore, irradiating the top of the square pillars with more energy leads to a more important ablation, explaining well the decrease in the square pillars’ height and increase in their width. The fact that the fluence only has a nuanced impact on the squares’ dimensions is intentional because they must be as similar as possible. Indeed, topographic changes affect the wetting behavior of the surface, which was not desired.

**Figure 8:**
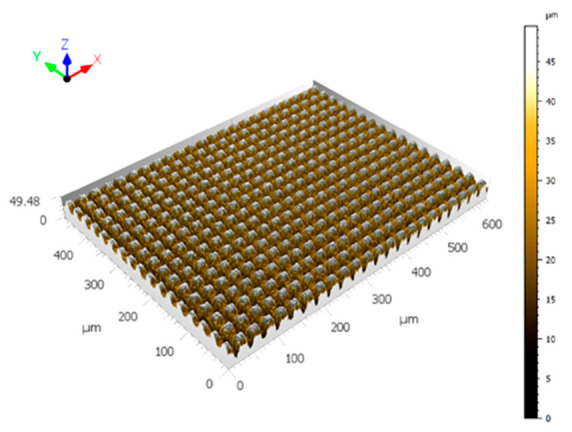
Interferometric image obtained with the MountainsMap software. This sample is the one for which the square pillars’ top was not irradiated.

**Figure 9:**
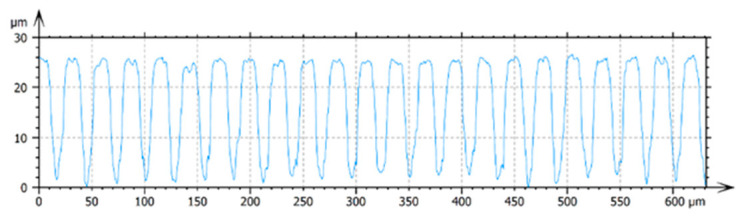
Unfiltered profile of the sample for which the square pillars’ top was not irradiated.

The arithmetic mean roughness, Ra, was calculated by extracting profiles from a selected area from the interferometric images (Figure 8). This area was chosen to be the top of the micrometric square pillars to limit the distorted values that could be obtained because of the high number of non-measured points on the slopes of the squares. Filtering was not applied. An example of the series of profiles obtained is provided in Figure 9.

**Figure 10:**
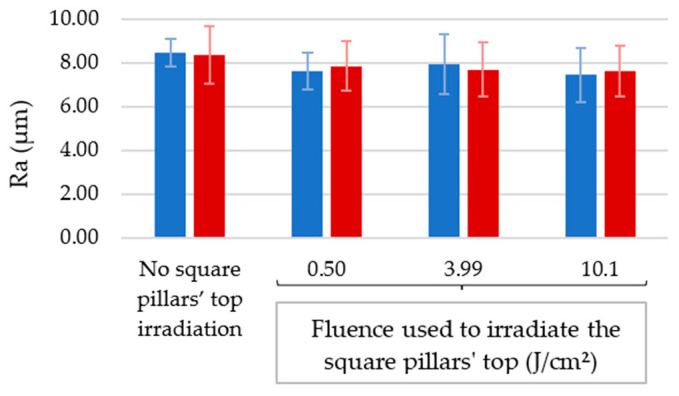
Arithmetic mean roughness (Ra) of the samples for which the square pillars’ top was irradiated using different fluences in ambient air or under a CO_2_ flow.

From Figure 10, we note that Ra values in the micrometric scale are similar independent of the fluence used to irradiate the square pillars’ top and of the texturing environment (8.1 ± 0.1 µm). We note a slight decrease with increasing fluence, which is consistent with the dimensions of the square pillars just detailed.

### 3.2. Effect of Texturing Process Environment on Wetting

**Figure 11:**
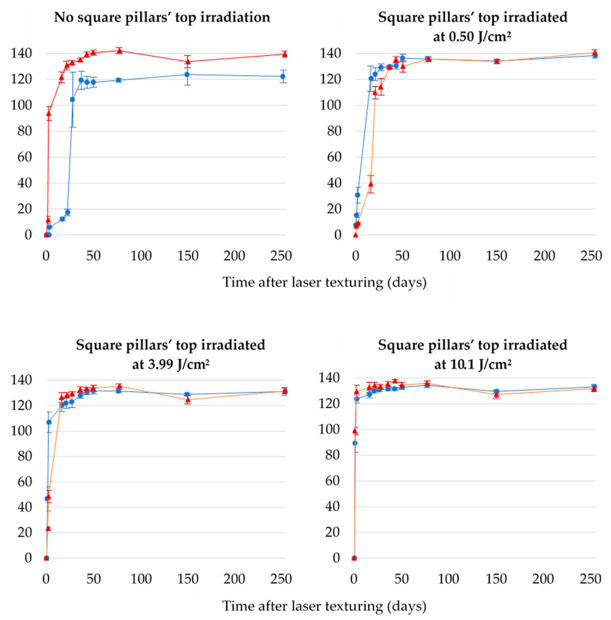
Evolution of water contact angle over time for samples textured in ambient air or under a CO_2_ flow using different fluences to irradiate the square pillars’ top.

Newly textured metallic surfaces are known to be superhydrophilic before transiting into a hydrophobic regime after some days [25,51,62,63,64,65]. To study the dependence of the texturing environment on this evolution, steady-state contact angle measurements were conducted for more than 250 days after laser processing for all the samples. The results are summarized in Table 1 and Table 2, and corresponding graphs are presented in Figure 11.

Figure 11 shows that the texturing environment has a greater impact on wetting evolution for the samples for which the square pillars’ top is not irradiated.

#### 3.2.1. Samples for Which the Square Pillars’ Top Was Not Irradiated

For these samples, the evolution of the steady-state contact angle is very different, whether the texturing process is performed in ambient air or under a CO_2_ flow. The surface switches from hydrophilicity to hydrophobicity much faster under CO_2_ than in ambient air. It takes 2 days when textured under the CO_2_ flow and in between 22 and 28 days in ambient air. However, stabilization of the steady-state contact angle occurs at about the same time for both samples: after about 37 days for the sample textured in ambient air and 43 days for the one textured under CO_2_. After 250 days, the sample textured under CO_2_ has a steady-state contact angle of 139 ± 2°, while that of the one textured in ambient air is equal to 122 ± 5°, making a difference of 17° (the difference is 10° when considering the standard deviation). When the square pillars’ top is not irradiated, a CO_2_ environment leads to a higher contact angle. On day 150, a slight temporary decrease in the steady-state contact angle is observed for the sample textured under CO_2_. This can be due to impurities adsorbed at the surface.

**Figure 12:**
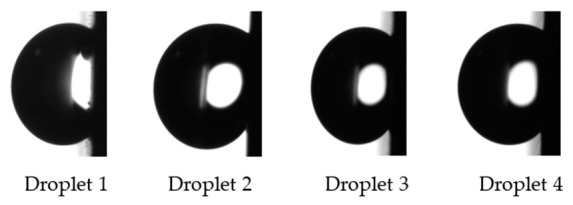
Droplets still present at 90° of inclination on the samples textured in ambient air.

The four droplets deposited on the samples textured in ambient air or under a CO_2_ flow were still on the surface at 90° of inclination. Figure 12 shows the droplets on the tilted sample textured in ambient air. Therefore, an authentic hysteresis cannot be measured using this method, and this is why, in this paper, we are mentioning θ_max_ − θ_min_ and not advancing and receding angles. This difference was calculated at 90° inclination to illustrate the droplet deformation upon tilt. A low value indicates that the droplet shape does not change when the surface is inclined.

For the sample textured in ambient air, θ_max_ − θ_min_ was equal to 12 ± 7°, and for the sample textured under the CO_2_, it was equal to 10 ± 2°. It is interesting to note that movements of the triple line were observed upon inclination for some droplets. For the sample textured in ambient air, the triple line moved at 49° and 30° of inclination. For the sample textured under a CO_2_ flow, it happened at 8°, 21°, 23°, and 37°.

Another method that can be employed to determine hysteresis is the volume-changing method. In this case, a droplet is deposited on the surface, and, after stabilization, the depositing needle is inserted in the droplet as close as possible to the substrate. It allows the addition of liquid, and when the contact line increases, the advancing angle is measured. After that, liquid can be extracted, and the receding angle can be measured when the contact line withdraws. This method has some drawbacks, including the interaction between the needle and the droplet that could alter the droplet’s behavior [66]. For these two samples, hysteresis was also measured using the volume-changing technique. The value θ_advancing_ – θ_receding_ was found to be equal to 109 ± 1° for the sample textured in ambient air, and 110 ± 1° for the one textured under CO_2_. These high hysteresis values confirm the high adhesion of the droplet.

Given the standard deviations, there is no significant difference in θ_max_ − θ_min_ and hysteresis values, whether the sample was textured in ambient air or under CO_2_. However, we can highlight that the triple line moves for every droplet deposited at lower inclination angles when the sample is textured under CO_2_.

#### 3.2.2. Samples for Which the Square Pillars’ Top Was Irradiated at Different Fluences

When the laser beam irradiates the top of the micrometric square pillars at different fluences, the evolution of the steady-state contact angle is remarkably similar regardless of the laser process environment, as shown in Figure 11. At 0.50 J/cm^2^, the sample textured in ambient air has a steady-state contact angle of 138 ± 2°, and for the sample textured under a CO_2_ flow, this angle is equal to 141 ± 2°. When higher fluences are used, the steady-state contact angles are smaller compared to the 0.50 J/cm^2^ fluence but identical in both environments considered: 131 ± 3° and 131 ± 2° for the samples whose tops were irradiated at 3.99 J/cm^2^ in ambient air and under a CO_2_ flow, respectively. For the 10.1 J/cm^2^ fluence, the sample textured in ambient air has a steady-state contact angle of 130 ± 1°, while that for the sample textured under a CO_2_ flow is equal to 132 ± 2°. The fact that higher fluence leads to smaller steady-state contact angles is contradictory to what can be found in the literature [23,55,67] but consistent with the topography of the corresponding samples. Indeed, in our case, with higher fluence, the square pillars are smoother with fewer ripples and, hence, lower steady-state contact angles, which are also reported by S. A. Jalil et al. [68] and others [69,70]. In agreement with the findings of Kietzig et al. [52], we found that, when the process was conducted under a CO_2_ flow, the average contact angle stabilized sooner than when the texturing process was carried out in ambient air. Furthermore, as can be seen in Figure 11 and Table 1 and Table 2, increasing the fluence leads to a faster wetting evolution. The average contact angle stabilized after 50 and 43 days for the samples having their square pillars’ tops irradiated at 0.50 J/cm^2^ in ambient air and under a CO_2_ flow, respectively. Considering the 3.99 J/cm^2^ fluence, the average contact angle stabilized after 43 and 36 days for the samples textured in ambient air and under a CO_2_ flow, respectively. For the 10.1 J/cm^2^ fluence, the stabilization of the average contact angle occurred after 21 and 16 days for the samples textured in ambient air and under a CO_2_ flow, respectively.

**Figure 13:**
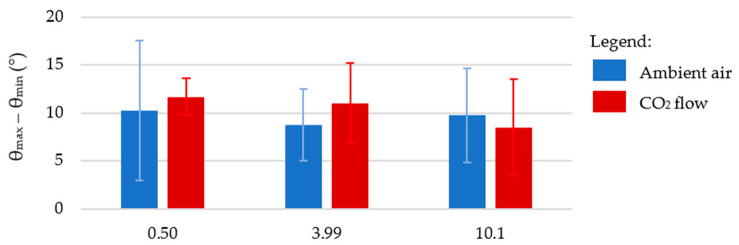
The θ_max_ − θ_min_ values for the samples for which the square pillars’ top was irradiated with different fluences, in ambient air or under a CO_2_ flow. These values were measured on day 252 and at 90° of surface inclination.

For these samples, the droplets were also present on the surface at 90° of inclination. The value θ_max_ − θ_min_ was quantified and found to be very similar in all cases, around 10°, as shown in Figure 13. The similarity of the θ_max_ − θ_min_ values, independent of the fluence used to irradiate the square pillars’ top, shows that θ_max_ − θ_min_ does not depend on the nanometric scale, as the nanometric asperities are the only topographic changes with increasing fluence.

The volume-changing technique was not used for these samples. As they show similar results in terms of steady-state contact angle and θ_max_ − θ_min_ (droplet high adhesion and negligible deformation upon inclination), comparable high hysteresis values are expected.

### 3.3. Wetting Regime and the Effect of Regular Wetting Measurements on Final Wettability

**Figure 14:**
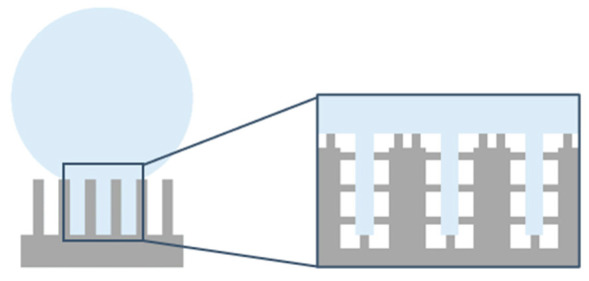
Rose petal configuration in samples textured with micrometric square pillars with nanometric roughness.

Our first hypothesis to explain the high water adhesion and minor droplet deformation upon the inclination of our surfaces is that we are probably in the so-called rose petal configuration [71], in which the nanometric scale of the surface is in the Cassie–Baxter regime (i.e., water not penetrating in the asperities), while the micrometric scale is in the Wenzel regime (i.e., micrometric asperities filled with water), as presented in Figure 14. Surfaces exhibiting the rose petal effect are known to have high hysteresis values [72]. Our results display high hysteresis values, high adhesion (droplets still on the surface at 90° of inclination), and negligible droplet deformation (low θ_max_ − θ_min_ at 90° of tilt). The high adhesion of the droplet can also be intensified by our specific topography, as we have cavities with depths ranging from 20 to 40 µm with aspect ratios above 1, leading to the anchorage of the droplet.

**Figure 15:**
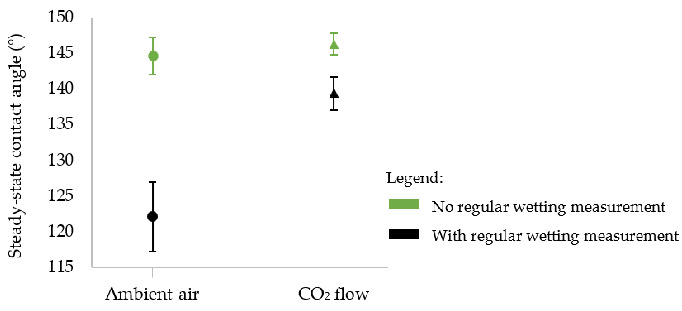
Steady-state contact angles of samples textured in ambient air or under a CO_2_ flow for which the square pillars’ top was not irradiated, and for which regular wetting measurements were (in black) or were not (in green) conducted over time.

To verify if performing wetting measurements over time can affect our final wettability and to try validating our hypothesis of the rose petal configuration of our droplets, we studied the effect of regular wetting measurements on steady-state contact angles and hysteresis. To that aim, two other samples without square pillars’ top irradiation were textured in ambient air and under a CO_2_ flow. They were stored in ambient air and not touched for more than 250 days. After this period, steady-state contact angles were measured and compared to the values obtained for the samples for which regular wetting over time was performed. The results are presented in Figure 15.

Figure 15 shows that when contact angles are not measured regularly, the steady-state contact angle is the same whether the sample is textured in ambient air or under a constant CO_2_ flow (145 ± 3° in ambient air vs. 146 ± 2° under CO_2_). Therefore, using a CO_2_ flow during laser processing did not lead to higher steady-state contact angles. This is in accordance with another study reported recently [73].

Figure 15 also demonstrates that performing wetting measurements on a chemically unstable surface alters the final wettability of the surface. When the sample is textured in ambient air, there is a significant difference of about 23° in the steady-state contact angle if it is regularly measured over time after laser texturing compared to when this contact angle evolution is not performed. This difference drops to 7° when the sample is textured under the CO_2_ flow, implying that the CO_2_ flow has a positive impact and minimizes the effect of regular wetting measurements.

Right after the laser texturing process, the surface is superhydrophilic due to the presence of polar iron and nickel–chromium oxides [52]. It becomes hydrophobic thanks to the adsorption of organic compounds [53] or the dissociative adsorption of carbon dioxide from ambient air [52,74,75]. These phenomena are possible thanks to the presence of oxygen-deficient oxides at the surface. Nonetheless, these oxygen-deficient oxides can also catalyze the dissociative adsorption of water to H^+^ and OH^−^ [76,77,78,79]. Moreover, S. Wippermann et al. [80] demonstrated that high water coverage favors the formation of complex structures composed of dissociated and undissociated water monomers, which could prevent the surface from reacting with favorable compounds to increase hydrophobicity. This possibility is very likely to happen when a droplet is deposited on a superhydrophilic and chemically unstable sample where the water penetrates its asperities and covers the entire surface.

**Figure 16:**
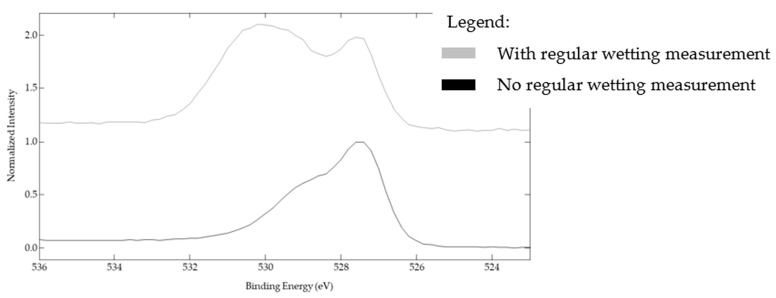
O1s spectra of the samples with no square pillars’ top irradiation textured under a CO_2_ flow and for which wetting evolution measurements were carried out (top spectrum—gray line) or were not carried out (bottom spectrum—black line).

To confirm the local surface chemistry changes, XPS analyses were carried out on the sample textured under the CO_2_ for which regular wetting measurements were and were not performed. Figure 16 illustrates how the high-resolution spectra of O1s are considerably different when the surface was regularly exposed to water.

**Figure 17:**
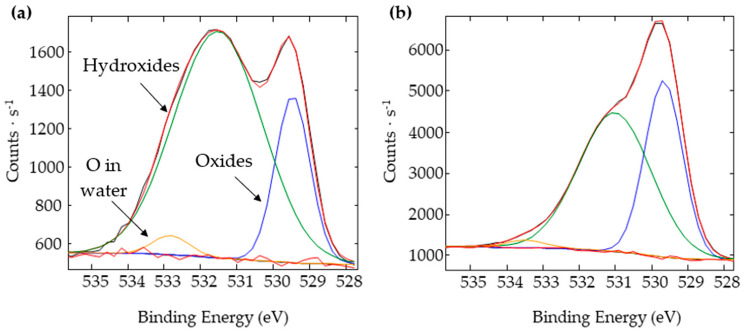
Deconvolution of the O1s spectra of the samples with no square pillars’ top irradiation textured under a CO_2_ flow for which wetting evolution measurements were (**a**) carried out and (**b**) not carried out.

When deconvoluting these spectra, three peaks can be detected (Figure 17a,b). The first peak at 529.5 eV corresponds to the oxides, the second one at about 531.5 eV corresponds to the oxygen in the hydroxyl group, and the third peak around 533 eV was fitted to be the oxygen in water.

The sample exposed to water has a much higher atomic percentage of hydroxyl groups (75% vs. 59% for the sample left undisturbed for 250 days) and a slight increase in the oxygen in water (3% vs. 2%) (Table 3). These results confirm that exposing our chemically unstable surfaces engenders surface chemistry changes by having more hydroxyl groups that lower the steady-state contact angle.

As mentioned, texturing under a CO_2_ flow is beneficial to reduce this influence of regular wetting measurements, and it can be explained by the high energy delivered by the laser during the texturing process. This high energy can activate its dissociative adsorption [52] and therefore prevent the dissociative adsorption of water as inactive stoichiometric magnetite is partially restored.

**Figure 18:**
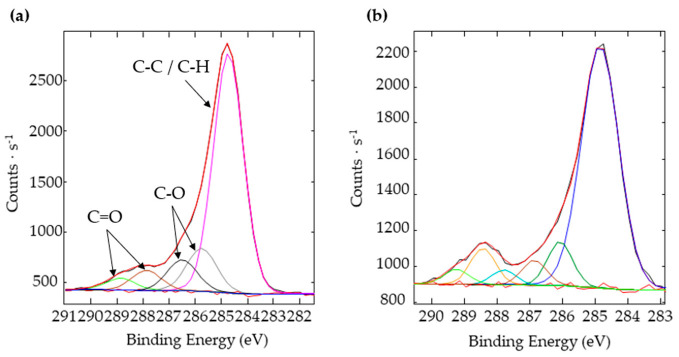
Deconvolution of the C1s spectra of the samples with no square pillars’ top irradiation textured under a CO_2_ flow for which wetting evolution measurements were (**a**) carried out and (**b**) not carried out.

High-resolution spectra of C1s were also recorded (Figure 18) to prove that a higher carbon content, whether coming from the dissociation of CO_2_ or the adsorption of organic compounds, would lead to higher steady-state contact angles. The samples considered for this analysis are the ones textured under the CO_2_ flow for which wetting evolution over time was and was not studied. Six peaks were distinguished on the C1s spectra: the peak at 284.8 eV corresponds to the C–C and C–H bonds, the peaks around 286 and 287 eV correspond to the C–O–H and C–O–C bonds, the peaks around 288 and 289 eV correspond to O–C=O and C=O bonds, and the peak with the highest bonding energy is attributed to carbonates.

We previously saw (Figure 15) that the surface not exposed to water during its chemical evolution presented a higher steady-state contact angle (146 ± 2° vs. 139 ± 2°). As can be calculated from Table 4, the ratio of non-polar bonds to polar bonds is higher when the surface has not been exposed to water: 3.2 vs. 2.3. This higher ratio means that the surface is mainly composed of non-polar bonds, which tends to make it more hydrophobic [25].

**Figure 19:**
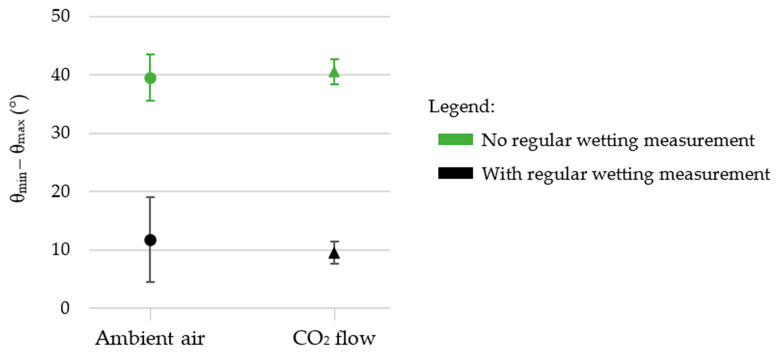
The θ_max_ − θ_min_ values for samples textured in ambient air or under a CO_2_ flow for which the square pillars’ top was not irradiated, and for which regular wetting measurements were (in black) or were not (in green) conducted over time.

Considering θ_max_ − θ_min_ (Figure 19), all the droplets deposited on the sample textured in ambient air and for which evolution of contact angle over time was not conducted were still present on the 90° tilted surface. The value θ_max_ − θ_min_ was found to be equal to 40 ± 4°. For the same sample textured under the CO_2_ flow, θ_max_ − θ_min_ was found to be equal to 41 ± 2°, which is in the range of the values found for the sample textured in ambient air. However, in the case of CO_2_, surprisingly, two droplets completely rolled off the surface at inclination angles of 46° and 50°. A true hysteresis could be measured and θ_advancing_ − θ_receding_ was found to be equal to 33 ± 4°. We make the hypothesis that for this sample, droplets are more likely to roll off unless impurities or water from ambient air are adsorbed at the surface.

**Figure 20:**
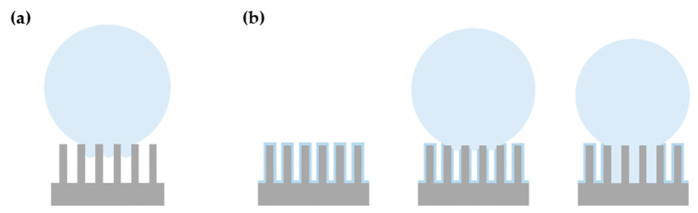
(**a**) First droplet deposited on the textured surface; (**b**) chemisorbed water on the surface and/or surface chemistry changes allowing anchorage of the next deposited droplet.

Figure 19 illustrates well the fact that these θ_max_ − θ_min_ values are higher than the ones obtained for the samples that were exposed to water. It means that the droplets’ shape is more deformed upon inclination. This and the fact that for one sample two droplets completely rolled off the surface upon inclination cancels the possibility of being in a rose petal configuration. Therefore, we suggest that the droplets deposited are always in an intermediate state where the droplet slightly penetrates the micrometric asperities, as illustrated in Figure 20a, as the droplets can adopt a different shape when submitted to gravity upon inclination. When regular wetting measurements are performed when the surface is chemically unstable, the droplets are also in an intermediate state, but local surface chemistry changes and chemisorbed water from the previous droplets make it possible for the next droplet deposited to anchor well on the surface, as illustrated in Figure 20b. This induces a smaller deformation of the droplet shape, and it can explain why we have low θ_max_ − θ_min_ values when wetting is conducted over time as the droplets are constrained in their initial position and shape.

Given the conditions at which these samples were textured, the use of CO_2_ during the laser process cannot be useful to obtain higher steady-state contact angles, and this is contrary to what can currently be found in the literature [52,73]. However, a study recently published presented similar results [73]. The CO_2_ flow seems to minimize the effect of regular wetting measurements by making inactive the most reactive sites of the surface right after or even during laser processing. This clarifies why the transition between the hydrophilic and hydrophobic regimes is observed sooner when the sample is textured under a CO_2_ flow.

## 4. Conclusions

In this work, we generated micrometric square pillars with a femtosecond laser in two different environments: in ambient air and under a CO_2_ flow. For some samples, the square pillars’ top was irradiated using different fluences. Our topographic analyses showed that the environment had no effect on the pillars’ dimensions and increasing fluences mainly affected the nanometric scale, as smoother areas and fewer ripples were spotted. Wetting measurements were carried out for more than 250 days and, interestingly, the environment had an effect on the samples for which the square pillars’ top was not irradiated. In this case, a CO_2_ environment allowed a quicker hydrophilic to hydrophobic transition (2 days vs. 22–28 days) and led to higher steady-state contact angles (139 ± 2° vs. 122 ± 5°). On the other hand, when the laser beam irradiated the top of the square pillars at different fluences, the environment had strictly no effect on steady-state contact angles, for which the values reached 140° for the lowest fluence used (0.50 J/cm^2^) and about 130° for the other fluences (3.99 J/cm^2^ and 10.1 J/cm^2^). In all cases, hysteresis could not be measured using the tilting method, as all the droplets were well anchored to the surfaces at 90° of inclination, and relatively low values of θ_max_ − θ_min_ were obtained (around 10°). Our first hypothesis was that our droplets were in a rose petal configuration. However, this was invalidated by the second part of our work, where we studied the effect of regular wetting measurements on final wettability. We showed that when no wetting measurements were performed over time, droplet deformation occurred upon the inclination of the surface, and θ_max_ − θ_min_ rose to about 40°. This suggested that our droplets for the undisturbed surfaces are in a unique intermediate state, i.e., water not fully penetrating the micrometric asperities. We also proved that performing regular wetting measurements when the surface is not chemically stable can drastically change the wetting behavior of a surface because of dissociative adsorption of water. Finally, we showed that texturing under a CO_2_ flow could not help reach higher steady-state contact angles (SSCA of 145° were found for the samples for which square pillars’ top was not irradiated, in ambient air and under the CO_2_ flow) but limited the effect of regular wetting measurements over time (due to local chemistry changes and/or water chemisorbed at the surface).

## Figures and Tables

**Table 1:** Wetting measurement results for the samples textured in ambient air.

Fluence Used to Irradiate the Top of the Square Pillars (J/cm^2^)	SSCA on Day 250	Number of Days for Stabilized SSCA	θ_max_ − θ_min_ at 90° of Inclination
None	122 ± 5°	37	12 ± 7°
0.50	138 ± 2°	50	10 ± 4°
3.99	131 ± 3°	43	9 ± 5°
10.1	130 ± 1°	21	10 ± 5°

**Table 2:** Wetting measurement results for the samples textured under a CO_2_ flow.

Fluence Used to Irradiate the Top of the Square Pillar (J/cm^2^)	SSCA on Day 250	Number of Days for Stabilized SSCA	θ_max_ − θ_min_ at 90° of Inclination
None	139 ± 2°	43	10 ± 2°
0.50	141 ± 2°	43	12 ± 4°
3.99	131 ± 2°	36	11 ± 5°
10.1	132 ± 2°	16	9 ± 2°

**Table 3:** Atomic percentages of the different oxygen bonds calculated from the O1s spectra of the samples with no square pillars’ top irradiation textured under a CO_2_ flow and for which wetting evolution measurements were and were not carried out.

Type of Bonding	Bonding Energy (eV)	At% with Regular Wetting Measurement	At% without Regular Wetting Measurement
Oxides	529.5	22%	39%
Hydroxides	531.5	75%	59%
Water	533	3%	2%

**Table 4:** Atomic percentages of the different carbon bonds calculated from the C1s spectra of the samples with no square pillars’ top irradiation textured under a CO_2_ flow and for which wetting evolution measurements were and were not carried out.

Type of Bonding	Bonding Energy (eV)	At% with Regular Wetting Measurement	At% without Regular Wetting Measurement
C–C/C–H	284.8	70%	76%
C–O–C/C–O–H	286	21%	14%
O–C=O/C=O	288	9%	10%

## Data Availability

All data are available within the manuscript.
